# Bis[2-(2-fur­yl)-1-(2-furylmeth­yl)-1*H*-benzimidazole-κ*N* ^3^]diiodidocadmium

**DOI:** 10.1107/S1600536811029321

**Published:** 2011-07-30

**Authors:** Huai-Xia Yang, Xia Wang, Cai-Xia Xie, Xiao-Fei Li, Yan-Ju Liu

**Affiliations:** a Pharmacy College, Henan University of Traditional Chinese Medicine, Zhengzhou 450008, People’s Republic of China

## Abstract

In the title complex, [CdI_2_(C_16_H_12_N_2_O_2_)_2_], the Cd^II^ atom is located on a twofold rotation axis and is four-coordinated by two N atoms from symmetry-related 2-(2-fur­yl)-1-(2-furyl­meth­yl)-1*H*-benzimidazole ligands and two I atoms in a distorted tetra­hedral configuration. The benzimidazole rings in adjacent mol­ecules are parallel, with an average inter­planar distance of 3.486 Å. The I atom is disordered over two sites in a 0.85 (5):0.15 (5) ratio.

## Related literature

For background to benzimidazole and its derivatives, see: Shi *et al.* (2010 ▶); Yang *et al.* (2008 ▶). For related structures containing cadmium, see: Wang *et al.* (2010 ▶); Zhai *et al.* (2006 ▶).
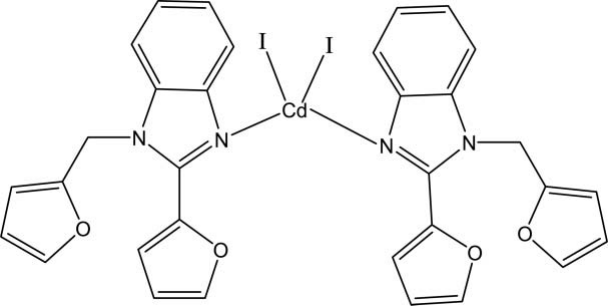


## Experimental

### 

#### Crystal data

[CdI_2_(C_16_H_12_N_2_O_2_)_2_]
*M* *_r_* = 894.75
Monoclinic, 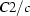

*a* = 18.140 (4) Å
*b* = 10.582 (2) Å
*c* = 18.507 (4) Å
β = 115.02 (3)°
*V* = 3219.2 (14) Å^3^
*Z* = 4
Mo *K*α radiation
μ = 2.64 mm^−1^
*T* = 293 K
0.18 × 0.16 × 0.15 mm

#### Data collection

Rigaku Saturn diffractometer
Absorption correction: multi-scan (*CrystalClear*; Rigaku/MSC, 2006 ▶) *T*_min_ = 0.648, *T*_max_ = 0.693
10971 measured reflections
2993 independent reflections
2595 reflections with *I* > 2σ(*I*)
*R*_int_ = 0.028

#### Refinement

*R*[*F* ^2^ > 2σ(*F* ^2^)] = 0.039
*wR*(*F* ^2^) = 0.085
*S* = 1.10
2993 reflections
205 parameters
H-atom parameters constrained
Δρ_max_ = 0.64 e Å^−3^
Δρ_min_ = −0.45 e Å^−3^

### 

Data collection: *CrystalClear* (Rigaku/MSC, 2006 ▶); cell refinement: *CrystalClear*; data reduction: *CrystalClear*; program(s) used to solve structure: *SHELXS97* (Sheldrick, 2008 ▶); program(s) used to refine structure: *SHELXL97* (Sheldrick, 2008 ▶); molecular graphics: *SHELXTL* (Sheldrick, 2008 ▶); software used to prepare material for publication: *SHELXTL*.

## Supplementary Material

Crystal structure: contains datablock(s) global, I. DOI: 10.1107/S1600536811029321/wm2505sup1.cif

Structure factors: contains datablock(s) I. DOI: 10.1107/S1600536811029321/wm2505Isup2.hkl

Additional supplementary materials:  crystallographic information; 3D view; checkCIF report

## Figures and Tables

**Fig. 1.**
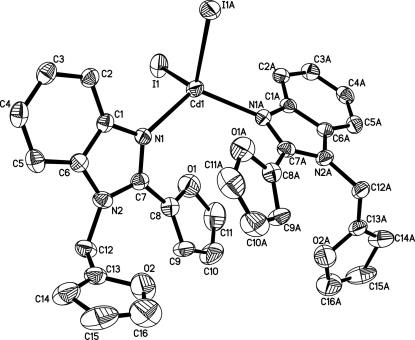
View of the title complex, showing the labeling of the 30% probability ellipsolids. For the disordered I atom, only one orientation is shown; H atoms have been omitted for clarity. [Symmetry code: A) x + 1, y, -z + 1/2]

**Fig. 2.**
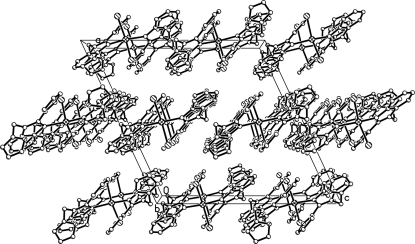
A view of the crystal packing along the b axis. H atoms are omitted for clarity.

## supplementary crystallographic information

### Comment

Benzimidazole and its derivatives have been used in the construction of complexes since they can act as polydentate ligands and function as bridging ligands (Yang *et al.*, 2008; Shi *et al.*, 2010). The Cd^II^ ion is a good model atom to construct complexes owing to its property to form bonds with different donors simultaneously (Zhai *et al.*, 2006; Wang *et al.*, 2010). In this work, synthesis and structure of the complex [Cd(C_16_H_12_N_2_O_2_)_2_I_2_] are described.

The Cd^II^ atom (site symmetry 2) is four-coordinated by two N atoms from two 2-(2-furyl)-1-(2-furylmethyl)-1*H*-benzimidazole ligands and two disordered I atoms in a distorted tetrahedral configuration (Fig. 1). The benzimidazole rings in adjacent molecules are parallel, with an average interplanar distance of 3.486 Å.

### Experimental

The ligand 2-(2-furyl)-1-(2-furylmethyl)-1*H*-benzimidazole (0.04 mmol) in methanol (6 ml) was added dropwise to a methanol solution (6 ml) of CdI_2_ (0.04 mmol) in methanol. The resulting solution was allowed to stand at room temperature. After two weeks light-yellow crystals with good quality were obtained and dried in air.

### Refinement

The disordered I atom was modelled by splitting the atom into two components (I1 and I1'), the site occupation factors of which refined in a ratio of 0.85 (5):0.15 (5). H atoms were positioned geometrically and refined as riding atoms, with C—H = 0.93 (aromatic) and 0.97 (CH_2_) Å, and with U_iso_(H) = 1.2 U_eq_(C).

### Crystal data

**Table:**

[CdI_2_(C_16_H_12_N_2_O_2_)_2_]	*F*(000) = 1720
*M**_r_* = 894.75	*D*_x_ = 1.846 Mg m^−^^3^
Monoclinic, *C*2/*c*	Mo *K*α radiation, λ = 0.71073 Å
Hall symbol: -C 2yc	Cell parameters from 4174 reflections
*a* = 18.140 (4) Å	θ = 2.3–27.9°
*b* = 10.582 (2) Å	µ = 2.64 mm^−^^1^
*c* = 18.507 (4) Å	*T* = 293 K
β = 115.02 (3)°	Prism, light yellow
*V* = 3219.2 (14) Å^3^	0.18 × 0.16 × 0.15 mm
*Z* = 4	

### Data collection

**Table:**

Rigaku Saturn diffractometer	2993 independent reflections
Radiation source: fine-focus sealed tube	2595 reflections with *I* > 2σ(*I*)
graphite	*R*_int_ = 0.028
Detector resolution: 28.5714 pixels mm^-1^	θ_max_ = 25.5°, θ_min_ = 2.3°
ω scans	*h* = −18→21
Absorption correction: multi-scan (*CrystalClear*; Rigaku/MSC, 2006)	*k* = −9→12
*T*_min_ = 0.648, *T*_max_ = 0.693	*l* = −22→22
10971 measured reflections	

### Refinement

**Table:**

Refinement on *F*^2^	Primary atom site location: structure-invariant direct methods
Least-squares matrix: full	Secondary atom site location: difference Fourier map
*R*[*F*^2^ > 2σ(*F*^2^)] = 0.039	Hydrogen site location: inferred from neighbouring sites
*wR*(*F*^2^) = 0.085	H-atom parameters constrained
*S* = 1.10	*w* = 1/[σ^2^(*F*_o_^2^) + (0.0357*P*)^2^ + 4.2457*P*] where *P* = (*F*_o_^2^ + 2*F*_c_^2^)/3
2993 reflections	(Δ/σ)_max_ = 0.001
205 parameters	Δρ_max_ = 0.64 e Å^−^^3^
0 restraints	Δρ_min_ = −0.45 e Å^−^^3^

### Special details

**Table:**

Geometry. All e.s.d.'s (except the e.s.d. in the dihedral angle between two l.s. planes) are estimated using the full covariance matrix. The cell e.s.d.'s are taken into account individually in the estimation of e.s.d.'s in distances, angles and torsion angles; correlations between e.s.d.'s in cell parameters are only used when they are defined by crystal symmetry. An approximate (isotropic) treatment of cell e.s.d.'s is used for estimating e.s.d.'s involving l.s. planes.
Refinement. Refinement of *F*^2^ against ALL reflections. The weighted *R*-factor *wR* and goodness of fit *S* are based on *F*^2^, conventional *R*-factors *R* are based on *F*, with *F* set to zero for negative *F*^2^. The threshold expression of *F*^2^ > σ(*F*^2^) is used only for calculating *R*-factors(gt) *etc*. and is not relevant to the choice of reflections for refinement. *R*-factors based on *F*^2^ are statistically about twice as large as those based on *F*, and *R*- factors based on ALL data will be even larger.

### Fractional atomic coordinates and isotropic or equivalent isotropic displacement parameters (Å^2^)

**Table:**

	*x*	*y*	*z*	*U*_iso_*/*U*_eq_	Occ. (<1)
Cd1	0.5000	0.58735 (4)	0.2500	0.04815 (16)	
I1	0.6326 (2)	0.7359 (4)	0.26829 (19)	0.0695 (6)	0.85 (5)
I1'	0.615 (5)	0.752 (5)	0.261 (2)	0.089 (7)	0.15 (5)
N1	0.4753 (2)	0.4731 (3)	0.1385 (2)	0.0429 (8)	
N2	0.4699 (2)	0.3215 (3)	0.0528 (2)	0.0483 (9)	
C1	0.4236 (2)	0.5121 (4)	0.0618 (2)	0.0426 (10)	
C2	0.3808 (3)	0.6239 (4)	0.0347 (3)	0.0491 (11)	
H2A	0.3830	0.6879	0.0700	0.059*	
C3	0.3349 (3)	0.6371 (5)	−0.0462 (3)	0.0555 (12)	
H3A	0.3060	0.7115	−0.0658	0.067*	
C4	0.3310 (3)	0.5417 (5)	−0.0990 (3)	0.0601 (13)	
H4A	0.2992	0.5534	−0.1532	0.072*	
C5	0.3730 (3)	0.4300 (5)	−0.0734 (3)	0.0552 (12)	
H5A	0.3706	0.3660	−0.1088	0.066*	
C6	0.4192 (2)	0.4180 (4)	0.0081 (3)	0.0439 (10)	
C7	0.5015 (2)	0.3599 (4)	0.1300 (3)	0.0459 (10)	
C8	0.5590 (3)	0.2866 (5)	0.1956 (3)	0.0529 (11)	
C9	0.5687 (3)	0.1627 (4)	0.2070 (3)	0.0599 (13)	
H9A	0.5385	0.1001	0.1716	0.072*	
C10	0.6321 (4)	0.1441 (7)	0.2812 (4)	0.092 (2)	
H10A	0.6531	0.0663	0.3038	0.111*	
C11	0.6575 (4)	0.2548 (8)	0.3144 (4)	0.098 (2)	
H11A	0.6989	0.2684	0.3649	0.118*	
C12	0.4852 (3)	0.2042 (4)	0.0182 (3)	0.0571 (12)	
H12A	0.5342	0.1643	0.0567	0.068*	
H12B	0.4940	0.2250	−0.0286	0.068*	
C13	0.4164 (3)	0.1140 (4)	−0.0046 (3)	0.0574 (12)	
C14	0.3614 (4)	0.0757 (5)	−0.0735 (4)	0.0829 (18)	
H14A	0.3571	0.0986	−0.1236	0.099*	
C15	0.3092 (4)	−0.0087 (6)	−0.0563 (5)	0.102 (2)	
H15A	0.2630	−0.0486	−0.0932	0.123*	
C16	0.3386 (5)	−0.0187 (7)	0.0207 (5)	0.109 (3)	
H16A	0.3171	−0.0691	0.0482	0.131*	
O1	0.6113 (3)	0.3501 (4)	0.2604 (3)	0.0905 (12)	
O2	0.4063 (3)	0.0562 (4)	0.0554 (3)	0.0995 (14)	

### Atomic displacement parameters (Å^2^)

**Table:**

	*U*^11^	*U*^22^	*U*^33^	*U*^12^	*U*^13^	*U*^23^
Cd1	0.0569 (3)	0.0407 (3)	0.0372 (2)	0.000	0.0104 (2)	0.000
I1	0.0791 (13)	0.0742 (10)	0.0499 (11)	−0.0321 (6)	0.0220 (8)	−0.0089 (5)
I1'	0.125 (16)	0.069 (7)	0.079 (7)	−0.051 (10)	0.049 (8)	−0.021 (5)
N1	0.048 (2)	0.040 (2)	0.0411 (19)	−0.0022 (16)	0.0185 (16)	−0.0024 (15)
N2	0.049 (2)	0.040 (2)	0.059 (2)	−0.0091 (17)	0.0264 (19)	−0.0103 (18)
C1	0.040 (2)	0.044 (2)	0.042 (2)	−0.0093 (19)	0.0159 (19)	0.0011 (19)
C2	0.055 (3)	0.043 (2)	0.050 (3)	−0.004 (2)	0.023 (2)	0.002 (2)
C3	0.051 (3)	0.057 (3)	0.048 (3)	−0.004 (2)	0.011 (2)	0.008 (2)
C4	0.059 (3)	0.074 (3)	0.041 (3)	−0.014 (3)	0.015 (2)	0.004 (2)
C5	0.054 (3)	0.065 (3)	0.046 (3)	−0.019 (2)	0.020 (2)	−0.014 (2)
C6	0.041 (2)	0.043 (2)	0.050 (2)	−0.0093 (19)	0.021 (2)	−0.002 (2)
C7	0.043 (2)	0.042 (2)	0.056 (3)	−0.0026 (19)	0.024 (2)	0.002 (2)
C8	0.049 (3)	0.057 (3)	0.053 (3)	−0.001 (2)	0.021 (2)	0.000 (2)
C9	0.070 (3)	0.033 (3)	0.067 (3)	0.003 (2)	0.020 (3)	−0.002 (2)
C10	0.089 (5)	0.081 (5)	0.102 (5)	0.038 (4)	0.036 (4)	0.037 (4)
C11	0.067 (4)	0.143 (7)	0.064 (4)	−0.003 (4)	0.006 (3)	0.015 (4)
C12	0.062 (3)	0.046 (3)	0.075 (3)	−0.011 (2)	0.041 (3)	−0.015 (2)
C13	0.067 (3)	0.040 (3)	0.076 (3)	−0.010 (2)	0.040 (3)	−0.011 (2)
C14	0.092 (4)	0.053 (3)	0.080 (4)	−0.014 (3)	0.013 (3)	0.006 (3)
C15	0.074 (4)	0.058 (4)	0.136 (7)	−0.025 (3)	0.006 (4)	−0.012 (4)
C16	0.108 (6)	0.095 (5)	0.139 (7)	−0.054 (5)	0.066 (6)	−0.031 (5)
O1	0.101 (3)	0.087 (3)	0.081 (3)	−0.009 (2)	0.036 (3)	−0.006 (2)
O2	0.121 (4)	0.099 (3)	0.091 (3)	−0.055 (3)	0.058 (3)	−0.031 (3)

### Geometric parameters (Å, °)

**Table:**

Cd1—N1	2.267 (3)	C7—C8	1.446 (6)
Cd1—N1^i^	2.267 (3)	C8—C9	1.328 (6)
Cd1—I1'^i^	2.67 (3)	C8—O1	1.351 (6)
Cd1—I1'	2.67 (3)	C9—C10	1.383 (8)
Cd1—I1^i^	2.772 (4)	C9—H9A	0.9300
Cd1—I1	2.772 (4)	C10—C11	1.312 (9)
N1—C7	1.323 (5)	C10—H10A	0.9300
N1—C1	1.393 (5)	C11—O1	1.419 (8)
N2—C7	1.356 (5)	C11—H11A	0.9300
N2—C6	1.390 (5)	C12—C13	1.482 (6)
N2—C12	1.476 (5)	C12—H12A	0.9700
C1—C6	1.386 (6)	C12—H12B	0.9700
C1—C2	1.387 (6)	C13—C14	1.307 (7)
C2—C3	1.379 (6)	C13—O2	1.348 (6)
C2—H2A	0.9300	C14—C15	1.432 (8)
C3—C4	1.386 (7)	C14—H14A	0.9300
C3—H3A	0.9300	C15—C16	1.297 (10)
C4—C5	1.378 (7)	C15—H15A	0.9300
C4—H4A	0.9300	C16—O2	1.371 (7)
C5—C6	1.387 (6)	C16—H16A	0.9300
C5—H5A	0.9300		
			
N1—Cd1—N1^i^	115.51 (17)	C1—C6—N2	106.2 (4)
N1—Cd1—I1'^i^	115.7 (13)	C5—C6—N2	131.2 (4)
N1^i^—Cd1—I1'^i^	105.3 (6)	N1—C7—N2	112.6 (4)
N1—Cd1—I1'	105.3 (6)	N1—C7—C8	123.5 (4)
N1^i^—Cd1—I1'	115.7 (13)	N2—C7—C8	123.8 (4)
I1'^i^—Cd1—I1'	98 (4)	C9—C8—O1	110.7 (5)
N1—Cd1—I1^i^	111.36 (12)	C9—C8—C7	131.5 (5)
N1^i^—Cd1—I1^i^	103.96 (12)	O1—C8—C7	117.7 (4)
I1'^i^—Cd1—I1^i^	6.6 (19)	C8—C9—C10	107.3 (5)
I1'—Cd1—I1^i^	104.6 (18)	C8—C9—H9A	126.4
N1—Cd1—I1	103.96 (12)	C10—C9—H9A	126.4
N1^i^—Cd1—I1	111.36 (12)	C11—C10—C9	108.6 (5)
I1'^i^—Cd1—I1	104.6 (18)	C11—C10—H10A	125.7
I1'—Cd1—I1	6.6 (18)	C9—C10—H10A	125.7
I1^i^—Cd1—I1	110.9 (2)	C10—C11—O1	108.6 (6)
C7—N1—C1	105.5 (3)	C10—C11—H11A	125.7
C7—N1—Cd1	130.5 (3)	O1—C11—H11A	125.7
C1—N1—Cd1	123.9 (3)	N2—C12—C13	112.1 (4)
C7—N2—C6	106.6 (3)	N2—C12—H12A	109.2
C7—N2—C12	129.5 (4)	C13—C12—H12A	109.2
C6—N2—C12	123.9 (4)	N2—C12—H12B	109.2
C6—C1—C2	120.0 (4)	C13—C12—H12B	109.2
C6—C1—N1	109.1 (4)	H12A—C12—H12B	107.9
C2—C1—N1	130.9 (4)	C14—C13—O2	110.4 (5)
C3—C2—C1	117.9 (4)	C14—C13—C12	132.9 (5)
C3—C2—H2A	121.1	O2—C13—C12	116.7 (5)
C1—C2—H2A	121.1	C13—C14—C15	106.3 (6)
C2—C3—C4	121.3 (5)	C13—C14—H14A	126.8
C2—C3—H3A	119.3	C15—C14—H14A	126.8
C4—C3—H3A	119.3	C16—C15—C14	107.0 (6)
C5—C4—C3	121.7 (4)	C16—C15—H15A	126.5
C5—C4—H4A	119.1	C14—C15—H15A	126.5
C3—C4—H4A	119.1	C15—C16—O2	109.7 (6)
C4—C5—C6	116.4 (4)	C15—C16—H16A	125.1
C4—C5—H5A	121.8	O2—C16—H16A	125.1
C6—C5—H5A	121.8	C8—O1—C11	104.8 (5)
C1—C6—C5	122.6 (4)	C13—O2—C16	106.5 (5)
			
N1^i^—Cd1—N1—C7	−29.6 (3)	Cd1—N1—C7—N2	177.3 (3)
I1'^i^—Cd1—N1—C7	−153.3 (16)	C1—N1—C7—C8	178.2 (4)
I1'—Cd1—N1—C7	99.3 (19)	Cd1—N1—C7—C8	−4.0 (6)
I1^i^—Cd1—N1—C7	−147.9 (3)	C6—N2—C7—N1	0.0 (5)
I1—Cd1—N1—C7	92.7 (4)	C12—N2—C7—N1	178.3 (4)
N1^i^—Cd1—N1—C1	147.9 (3)	C6—N2—C7—C8	−178.7 (4)
I1'^i^—Cd1—N1—C1	24.3 (16)	C12—N2—C7—C8	−0.4 (7)
I1'—Cd1—N1—C1	−83.1 (19)	N1—C7—C8—C9	149.8 (5)
I1^i^—Cd1—N1—C1	29.6 (3)	N2—C7—C8—C9	−31.7 (8)
I1—Cd1—N1—C1	−89.8 (3)	N1—C7—C8—O1	−26.6 (6)
C7—N1—C1—C6	0.9 (4)	N2—C7—C8—O1	152.0 (4)
Cd1—N1—C1—C6	−177.2 (3)	O1—C8—C9—C10	−2.2 (6)
C7—N1—C1—C2	−178.1 (4)	C7—C8—C9—C10	−178.7 (5)
Cd1—N1—C1—C2	3.9 (6)	C8—C9—C10—C11	2.2 (7)
C6—C1—C2—C3	−0.2 (6)	C9—C10—C11—O1	−1.3 (8)
N1—C1—C2—C3	178.7 (4)	C7—N2—C12—C13	104.9 (5)
C1—C2—C3—C4	0.4 (7)	C6—N2—C12—C13	−77.1 (5)
C2—C3—C4—C5	−0.4 (7)	N2—C12—C13—C14	109.2 (7)
C3—C4—C5—C6	0.2 (7)	N2—C12—C13—O2	−71.6 (6)
C2—C1—C6—C5	0.0 (6)	O2—C13—C14—C15	3.2 (7)
N1—C1—C6—C5	−179.1 (4)	C12—C13—C14—C15	−177.6 (5)
C2—C1—C6—N2	178.2 (4)	C13—C14—C15—C16	−2.7 (8)
N1—C1—C6—N2	−0.9 (4)	C14—C15—C16—O2	1.2 (9)
C4—C5—C6—C1	0.0 (6)	C9—C8—O1—C11	1.4 (6)
C4—C5—C6—N2	−177.7 (4)	C7—C8—O1—C11	178.5 (5)
C7—N2—C6—C1	0.6 (4)	C10—C11—O1—C8	0.0 (7)
C12—N2—C6—C1	−177.8 (4)	C14—C13—O2—C16	−2.5 (7)
C7—N2—C6—C5	178.6 (4)	C12—C13—O2—C16	178.2 (5)
C12—N2—C6—C5	0.2 (7)	C15—C16—O2—C13	0.6 (8)
C1—N1—C7—N2	−0.5 (4)		

Symmetry codes: (i) −*x*+1, *y*, −*z*+1/2.

## References

[bb1] Rigaku/MSC (2006). *CrystalClear* Rigaku Americas Corporation, The Woodlands, Texas, USA.

[bb2] Sheldrick, G. M. (2008). *Acta Cryst.* A**64**, 112–122. 10.1107/S0108767307043930 18156677

[bb3] Shi, X.-J., Wang, X., Li, L.-K., Hou, H.-W. & Fan, Y.-T. (2010). *Cryst. Growth Des.* **10**, 2490–2500.

[bb4] Wang, X., Li, Y.-X., Liu, Y.-J., Yang, H.-X. & Zhang, C.-C. (2010). *Acta Cryst.* E**66**, m1207. 10.1107/S1600536810034409 PMC2983315 21587366

[bb5] Yang, H.-X., Meng, X.-R., Liu, Y., Hou, H.-W., Fan, Y.-T. & Shen, X.-Q. (2008). *J. Solid State Chem.* **181**, 2178–2184.

[bb6] Zhai, Q.-G., Wu, X.-Y., Chen, S.-M., Lu, C.-Z. & Yang, W.-B. (2006). *Cryst. Growth Des.* **6**, 2126–2135.

